# Morin decreases galectin-3 expression and sensitizes ovarian cancer cells to cisplatin

**DOI:** 10.1007/s00404-018-4912-4

**Published:** 2018-09-28

**Authors:** Dominik Bieg, Daniel Sypniewski, Ewa Nowak, Ilona Bednarek

**Affiliations:** Department of Biotechnology and Genetic Engineering, School of Pharmacy with the Division of Laboratory Medicine in Sosnowiec, Medical University of Silesia, Katowice, Poland, Jedności Street 8, 41-200 Sosnowiec, Poland

**Keywords:** Ovarian cancer, Drug resistance, Cisplatin, Morin, Galectin-3

## Abstract

**Purpose:**

This study aimed at evaluating whether morin (a natural flavonoid and a known inhibitor of NF-κB) can sensitize ovarian cancer cells to cisplatin by decreasing the expression of galectin-3, which is an anti-apoptotic protein regulated by NF-κB transcription factor.

**Methods:**

To assess the possibility of augmentation the activity of cisplatin by morin, we studied the separate and the combined effect of morin and cisplatin on viability, proliferation, and apoptosis of TOV-21G (cisplatin-sensitive) and SK-OV-3 (cisplatin-resistant) ovarian cancer cells. We also analysed the effect of morin and cisplatin on galectin-3 expression at the mRNA and protein levels.

**Results:**

We demonstrated that morin possess antitumor activity against TOV-21G and SK-OV-3 ovarian cancer cells by reducing cell viability and proliferation as well as increasing the induction of apoptosis. Co-treatment of the cells with selected concentrations of morin and cisplatin, accordingly to specific treatment approaches, reveals a synergism, which leads to sensitization of the cells to cisplatin. During this sensitization, morin significantly reduces the expression of galectin-3 at the mRNA and protein level, regardless of the presence of cisplatin.

**Conclusions:**

Morin sensitizes TOV-21G and SK-OV-3 ovarian cancer cells to cisplatin, what is associated with a decrease of the expression of galectin-3.

## Introduction

Ovarian cancer is one of the leading causes of cancer death in female worldwide [1–5], despite the fact that it accounts for only 3% of all cancer in women [6]. Cytoreductive surgery followed by treatment with combination of cisplatin (or carboplatin) and paclitaxel is currently the standard method for ovarian cancer therapy [3–5]. The two main reasons of high mortality in ovarian cancer are lack of early symptoms and platinum resistance of cancer cells (intrinsic or acquired). Due to the difficulties in detection, most patients are diagnosed at an advanced stage in which often occurs intrinsic platinum resistance [1]. Even if the initial treatment is successful, it often ensues recurrence with acquired resistance to further chemotherapy [2]. Resistance to platinum at the beginning of treatment or at relapse is the most significant cause of ovarian cancer patient’s death and continues to remain a major problem [1, 2]. Developing of new effectual alternative modulators of platinum agents, to effectively overcome resistance, is becoming an urgent need [3, 4].

The anticancer effect of cisplatin (*cis*-diamminedichloroplatinum(II)) involves active uptake into cells, followed by forming DNA adducts, that cause single or double-strand DNA breaks. The DNA damages result in DNA replication and cell cycle arresting, as well as activation of the cellular apoptosis [7, 8]. Cisplatin resistance cannot be explained by a simple mechanism. One of its underlying factors is an impairment of apoptosis, involving the altered expression of proteins such as BCL-2 family members and defects in several signal transducers, including NF-κB [9, 10]. It is reported that NF-κB can also influence the expression of BCL-2 proteins and that constitutive NF-κB activation has been observed in many cancers resistant to antitumor agents (including SK-OV-3 ovarian cancer cells) [10, 11]. Moreover, NF-κB inhibitors enhance cisplatin’s antitumor capabilities against some cisplatin-resistant cell lines (including ovarian cancer) [10, 12]. Another protein (which has been increasingly associated in the literature with the cancer resistance, due to its structure similarity to BCL-2 family members and its regulation by NF-κB) is galectin-3 [13].

Galectin-3 (coded by *LGALS3* gene), a chimera-type member of β-galactose-binding protein family, is a multifunctional glycoprotein associated with cell growth, differentiation, adhesion, migration, apoptosis, metastasis, neoplastic transformation, and angiogenesis [5, 14–16]. Galectin-3 in cytoplasm is a well-known anti-apoptotic agent [17]. It contains the NWGR (N, asparagine; W, tryptophan; G, glycine; R, arginine) anti-death motif, which is specific for the BCL-2 family and is resposible for an anti-apoptotic activity of galectin-3 and BCL-2 [16, 18]. It has been shown in several types of cancer that in response to chemotherapeutic agents (such as cisplatin, etoposide, Tumour Necrosis Factor-α (TNF-α), and nitric oxide), galectin-3 is transported from the nucleus to the cytoplasm, where it stimulates the phosphorylation of Bcl-2 associated death (Bad) protein and the reduction of Bad expression. This results in the stabilization of mitochondrial membrane integrity, and subsequently it blocks cytochrome c release, caspase-3 activation, and finally inhibits apoptosis [15–18]. Galectin-3 expression is regulated by NF-κB since its promoter region contains two NF-κB-like sites [13]. According to published data, the overexpression of galectin-3 occurs in cancers of tongue, thyroid, colon, liver, gastric, hepatocellular, and ovaries. Furthermore, up-regulation of galectin-3 in various cancer cells (including ovarian cancer) makes them resistant to chemotherapeutic treatment [5, 15–18].

Since chemoresistance is one of the most significant problems in ovarian cancer treatment, many studies focus on plant-derived bioactive compounds, which could sensitize cancer cells to cisplatin [10]. One of these natural compounds is morin (3,5,7,2′,4′-pentahydroxyflavone), a flavonoid originally isolated from *Morus alba* L (white mulberry) and widely distributed in fruits such as fig, almond, sweet chestnut, and old fustic [19–21]. Morin exhibits various biological properties such as anti-inflammatory (inhibition of cytokines release), anti-oxidative (xanthine oxidase inhibitor property, prevention of low-density lipoprotein oxidation, free radical scavenging activity), anti-mutagenic (protective effect against DNA damage caused by free radical) [7, 19]. Increasing evidences also reveal an anti-cancer potential of morin through inhibiting proliferation and promoting apoptosis and chemo-sensitivity of various cancer cell lines [19–21]; however, until now there has been no research on the use of morin in ovarian cancer. The antitumor effect of morin is achieved by suppressing the activation of NF-κB, what consequently inhibits expression of the genes regulated by this factor [19, 20].

In view of the fact that morin is a known inhibitor of NF-κB, which in turn may influence the expression of galectin-3 (the anti-apoptotic protein), we hypothesized that morin will sensitize ovarian cancer cells to cisplatin, what will be achieved by reducing the expression of galectin-3.

## Materials and methods

### Cell culture and drugs

SK-OV-3 human ovarian cancer (adenocarcinoma) cells from American Type Culture Collection (ATCC^®^ HTB-77™) were cultured in RPMI-1640 medium (Lonza) supplemented with 10% (v/v) FBS (foetal bovine serum; Gibco^®^) and 50 µg/ml gentamycin (Biological Industries). TOV-21G human ovarian cancer (grade 3, stage III, primary malignant adenocarcinoma; clear cell carcinoma) cells from American Type Culture Collection (ATCC^®^ CRL-11730™) were grown in the mixture (1:1) of MCDB-105 medium (Biological Industries) and M-199 Earle’s Salts Base medium (Biological Industries) supplemented with 15% (v/v) FBS (Gibco^®^) and 50 µg/ml gentamycin (Biological Industries). Both cell lines were cultivated at 37 °C in a humidified atmosphere of 95% air and 5% CO_2_.

Morin was obtained from Sigma-Aldrich, dissolved in DMSO (dimethyl sulfoxide; BioShop Canada Inc.) at a concentration of 50 mM and stored in – 20 °C. Cisplatin was acquired from Sigma-Aldrich, dissolved in 0.9% NaCl solution (Polpharma) at a concentration of 1 mg/ml (3333 mM), and stored in − 20 °C.

### Cell viability assay

Cell viability assay was performed using: XTT (2,3-Bis(2-methoxy-4-nitro-5-sulfophenyl)-2H-tetrazolium-5-carboxanilide inner salt; BioShop Canada Inc.) dissolved in phosphate-buffered saline (PBS) solution (Gibco^®^); phenazine methosulfate (PMS) solution (Promega); and RPMI-1640 medium without phenol red (Gibco^®^).

For the XTT assay, cells were seeded at 6 × 10^3^ cells/100 µl medium/0.32 cm^2^ growth area in 96-well plates, grown overnight, and treated with morin or cisplatin for 24 h and/or 48 h. Concentrations of drugs’ solvents were corrected in all wells (including control wells) to the constant level, corresponding to the highest used concentration of a particular solvent. Following the treatments, the medium in each well was replaced with 100 µl of the mixture of RPMI-1640 medium without phenol red, XTT solution (at the final concentration of 200 µg/ml) and PMS solution (at the final concentration of 2 µg/ml), prior to incubation at 37 °C for 3 h in the dark. The absorbance of each well was measured at 450 nm with ELISA plate reader (Dynex Technologies Triad Multi-Mode Microplate Reader). All treated cells were compared against control cells (considered as 100% viable). IC_50_ (half maximal inhibitory concentration) values were determined for each drug at each treatment time. Samples were prepared in triplicate.

### Cell proliferation assay

For the EdU (5-ethynyl-2′-deoxyuridine) incorporation assay, cells were seeded at 18 × 10^3^ cells/250 µl medium/0.95 cm^2^ growth area in 48-well plates, grown overnight, and treated with morin or cisplatin for 24 h and/or 48 h. Concentrations of drugs’ solvents were corrected in all wells (including control wells) to the constant level, corresponding to the highest used concentration of a particular solvent. After the treatments, the cell proliferation assay was performed using Click-iT^®^ EdU Imaging Kit (Invitrogen™) according to the manufacturer’s protocol. Additionally, the nuclei of the examined cells were visualised by staining for 5 min in DAPI (4′,6-diamidino-2-phenylindole dihydrochloride; Sigma-Aldrich) at the final concentration of 5 µg/ml. The cells were counted with a fluorescence microscope Eclipse Ti (Nikon Instruments Inc.) For each well, the ratio of proliferating cells to the total number of cells in three different fields was calculated and the results were compared against control cells (considered as 100% proliferating). GI_50_ (half maximal growth inhibitory concentration) values were determined for each drug at each treatment time. Samples were prepared in triplicate.

### Apoptosis assay

For the apoptosis assay, cells were seeded at 18 × 10^3^ cells/250 µl medium/0.95 cm^2^ growth area in 48-well plates, grown overnight, and treated with morin or cisplatin for 24 h and/or 48 h. Concentrations of drugs’ solvents were corrected in all wells (including control wells) to the constant level, corresponding to the highest used concentration of a particular solvent. After the treatments, the assay was performed using FITC Annexin V/Dead Cell Apoptosis Kit with FITC annexin V and PI (Invitrogen™) according to the manufacturer’s protocol. The cells were counted with a fluorescence microscope Eclipse Ti (Nikon Instruments Inc.) For each well, the ratio of apoptotic cells to the total number of cells in three different fields was calculated. Samples were prepared in triplicate.

### Drug combination studies

Evaluation of the combined effects of morin with cisplatin on cell viability, proliferation or apoptosis was performed exactly as described for each of these assays individually; however, the drugs were combined (in a non-constant ratio), rather than used separately. Briefly, the selected concentrations of morin were mixed with the selected concentrations of cisplatin. Concentrations of drugs’ solvents were corrected in all wells (including control wells) to the constant level, corresponding to the highest used concentration of a particular solvent. The nature of the interaction between morin and cisplatin was assessed using different approaches: (APP:1) simultaneous treatment with both drugs for 24 h; and/or (APP:2) pre-treatment with morin for 24 h, followed by treatment with cisplatin alone for the another 24 h; and/or (APP:3) pre-treatment with morin for 24 h, followed by co-treatment with morin and cisplatin for another 24 h. The cytotoxic, anti-proliferative, and pro-apoptotic effect of morin–cisplatin combination against cancer cells over a range of concentrations was compared to those obtained for the individual drugs. A measure of the synergy between the two drugs, referred to as the combination index (CI), was calculated using CompuSyn software (ComboSyn, Inc.) developed on the basis of median effect mathematical algorithm [22]. The following assumption was made: a drug combination was synergistic if its CI value was below 0.9; the combination was additive when the CI was between 0.9 and 1.1; and the combination was antagonistic as indicated by CI values above 1.1 [23].

### Real-Time™ RT-PCR analysis

Total RNA was extracted from cells seeded at 3.75 × 10^4^ cells/500 µl medium/2 cm^2^ growth area in 24-well plates, grown overnight, and treated with morin and/or cisplatin for 24 h and/or 48 h. Concentrations of drugs’ solvents were corrected in all wells (including control wells) to the constant level, corresponding to the highest used concentration of a particular solvent. The isolation procedure was performed using Direct-zol™ RNA MiniPrep (ZymoResearch) supplemented with TRI Reagent™ Solution (Invitrogen™) according to the manufacturer’s protocol. Extracts were quantified spectrophotometrically by BioPhotometer (Eppendorf) equipped with µCuvette^®^ G1.0 (Eppendorf), and their quality and integrity were also verified by 1% agarose gel electrophoresis.

The Real-Time™ RT-PCR was performed on the Stratagene Mx3000P Instrument using Brilliant II SYBR^®^ Green QRT-PCR Master Mix Kit (Agilent Technologies). The reagent mixture was prepared according to the instruction manual with the final primers’ concentration of 0.2 µM and the final template concentration of 8 ng/µl. The reverse transcription step was performed at 55 °C for 30 min. This was followed by initial denaturation at 95 °C for 10 min; 40 cycles of denaturation (94 °C, 15 s), annealing (63 °C, 60 s, with fluorescence measurement at the endpoint), and extension (72 °C, 30 s); and a final extension step at 72 °C for 10 min. The dissociation curves were generated by incubating the amplicons for 1 min at 95 °C, ramping down to 65 °C, and then increasing the temperature to 95 °C at a rate of 0.5 °C/s and fluorescence measuring at the all points. To confirm the specificity, amplification products were additionally electrophoresed on 3% agarose gels. The expression of galectin-3 was quantified using the comparative method ($2^{{ - \Delta \Delta C_{\text{q}} }}$). The reference gene, chosen for normalization, was *TATA*-*box binding protein* (*TBP*) housekeeping gene. The following primers were designed with the *Primer*-*BLAST* (NCBI) and used for Galectin-3: 5′-GCC AAC GAG CGG AAA ATG G-3′ (forward), 5′-TCC TTG AGG GTT TGG GTT TCC-3′ (reverse), and for *TBP*: 5′-TAT AAT CCC AAG CGG TTT GCT G-3′ (forward), 5′-GCC AGT CTG GAC TGT TCT TCA-3′ (reverse). All primers were synthesized by Institute of Biochemistry and Biophysics, Polish Academy of Sciences in Warsaw. Samples were prepared in triplicate and fluorescence measurement for each sample was done in duplicate.

### ELISA assay

For the assay, cells were seeded at 1.8 × 10^5^ cells/1 ml medium/3.8 cm^2^ growth area in 12-well plates, grown overnight, and treated with morin and/or cisplatin for 24 h and/or 48 h. Concentrations of drugs’ solvents were corrected in all wells (including control wells) to the constant level, corresponding to the highest used concentration of a particular solvent. Total protein extraction and ELISA assay were performed using Galectin-3 Human SimpleStep ELISA^®^ Kit (Abcam) and the concentrations of proteins in solutions were determined using Bradford Reagent (Sigma-Aldrich) and Bovine Serum Albumin Standard Set (Fermentas) according to the manufacturers’ protocols. The absorbance of each well was measured at 450 nm (ELISA assay) or 595 nm (Bradford assay) with ELISA plate reader (Dynex Technologies Triad Multi-Mode Microplate Reader). The concentration of galectin-3 in 1 µg of protein extract in each sample was determined by interpolating the blank control subtracted absorbance values against the standard curve and by multiplying the resulting value by the appropriate sample dilution factor. Samples were prepared in triplicate and absorbance measurement for each sample was done in duplicate.

### Statistical analysis

Differences between the groups were tested using Statistica 12.5 (StatSoft) by a *t* test for independent samples or a one-way or a two-way analysis of variance (ANOVA) followed by post hoc Tukey’s HSD test. The assumptions of normal distribution of variables and homogeneity of variances in each group were tested using the Shapiro–Wilk test and the Levene’s test, respectively. Data are presented as the mean ± standard deviation (SD). A *p* values less than 0.05 were considered as significant.

## Results

### Morin enhances the cytotoxicity of cisplatin against ovarian cancer cells

**Fig. 1:**
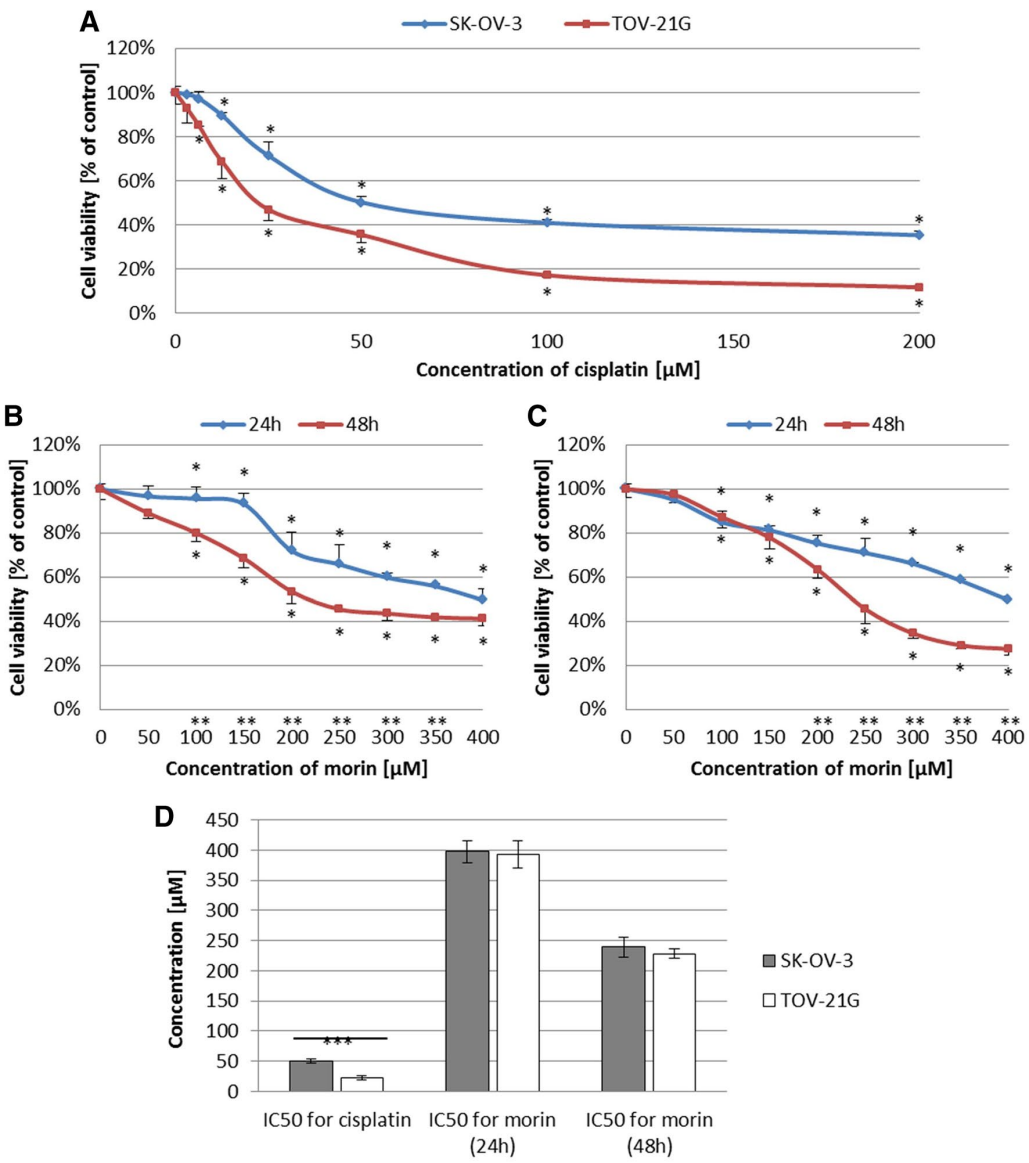
Effects of morin and cisplatin on viability of TOV-21G (cisplatin-sensitive) and SK-OV-3 (cisplatin-resistant) human ovarian cancer cells. The cells were treated with indicated concentrations of the drugs in indicated periods of time: **a** TOV-21G and SK-OV-3 cells treated with cisplatin (3.125–200 µM) for 24 h, **b** TOV-21G cells treated with morin (50–500 µM) for 24 h and 48 h, **c** SK-OV-3 cells treated with morin (50–500 µM) for 24 h and 48 h. Cell viability was analysed by XTT assay. The data are shown as mean ± SD of triplicate experiments. Asterisk: ANOVA *p* < 0.05 between the cells treated with the different concentrations of drug and the untreated control group in the same period of time (dose-dependence). Double asterisk: ANOVA *p* < 0.05 between the cells treated with the same concentration of drug in the different period of time (time-dependence). The cytotoxicity of morin was dose- and time-dependent, and cisplatin was dose-dependent. **d** Comparison of IC_50_ values obtained for TOV-21G and SK-OV-3 cells treated with morin for 24 h and 48 h or cisplatin for 24 h. Triple asterisk: *T* test *p* < 0.05 between IC_50_ values obtained for the same drug in the same period of time in different cell lines (TOV-21G or SK-OV-3)

To investigate whether morin can modulate the cytotoxic effect of cisplatin against ovarian cancer cells, we first evaluated the viability of TOV-21G and SK-OV-3 cells treated with cisplatin (3.125–200 µM) for 24 h or morin (50–400 µM) for 24 h and 48 h. The viability of both cell lines was inhibited by cisplatin in a dose-dependent manner (both *p* < 0.001; Fig. 1a). However, the calculated IC_50_ values (IC_50_ = 50.05 ± 3.01 µM for SK-OV-3 and IC_50_ = 22.96 ± 3.22 µM for TOV-21G) were statistically different (*p* < 0.001; Fig. 1d). Furthermore, morin treatment also revealed its cytotoxic effect in a dose- and time-dependent manner (TOV-21G *p* < 0.01; SK-OV-3 *p* < 0.001; Fig. 1b, c), but there were no significant differences between IC_50_ values obtained for both cell lines during 24-h treatment (IC_50_ = 392.59 ± 22.55 µM and 397.85 ± 18.54 µM for TOV-21G and SK-OV-3, respectively; Fig. 1d) or 48-h treatment (IC_50_ = 228.52 ± 7.77 µM and 239.09 ± 16.59 µM for TOV-21G and SK-OV-3, respectively; Fig. 1d).

**Fig. 2:**
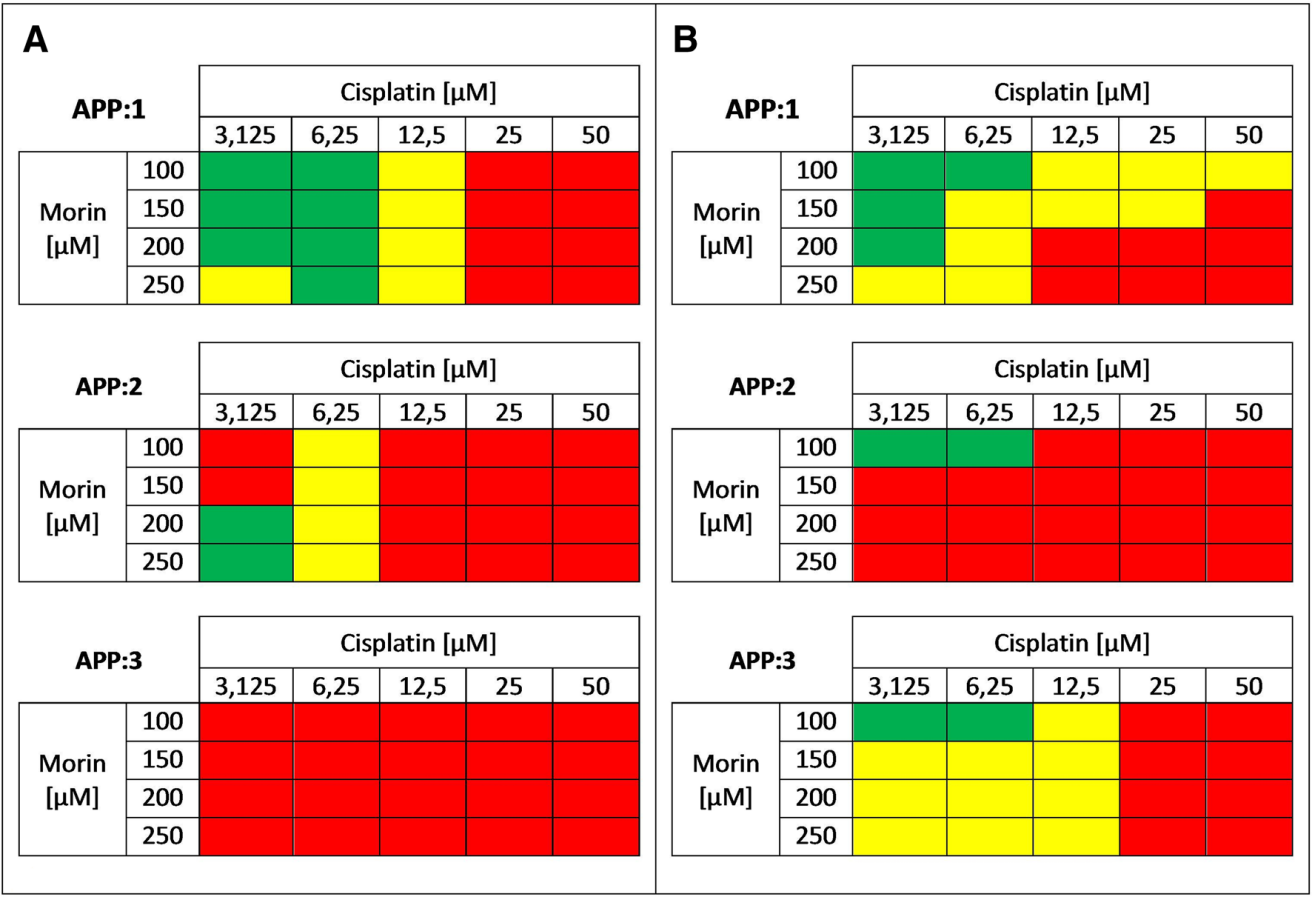
Cytotoxic effect of morin-cisplatin combination on **a** TOV-21G (cisplatin-sensitive) and **b** SK-OV-3 (cisplatin-resistant) human ovarian cancer cells. The cells were treated with different combinations of morin–cisplatin according to three approaches: APP:1—simultaneous treatment with both drugs for 24 h; APP:2—pre-treatment with morin for 24 h, followed by treatment with cisplatin alone for the another 24 h; APP:3—pre-treatment with morin for 24 h, followed by co-treatment with morin and cisplatin for another 24 h. After the treatments the cell viability was determined (XTT assay), followed by the calculation of combination index (CI). The CI values are shown as heat maps, where the effect of a drug combination is synergistic if CI < 0.9 (green colour); additive if 0.9 ≤ CI ≤ 1.1 (yellow colour); antagonistic if CI > 1.1 (red colour)

Next, we performed morin–cisplatin combination cytotoxicity study to evaluate interactions between morin and cisplatin and to choose the most effective doses of both drugs for further steps of this experiment. The range of concentrations for the assessment was selected based on an approximate cytotoxicity of 30–50%, when the agents were used alone. All *r* values calculated by CompuSyn software (ComboSyn, Inc.) were greater than 0.95, as it was described for the cell culture experiments [1, 24]. The results are shown as a heat map in Fig. 2.

When TOV-21G cells were treated with morin and cisplatin simultaneously for 24 h (APP:1; Fig. 2a), there occurred a synergism at low concentrations of cisplatin (3.125–6.25 µM), an additive effect at a medium dose of cisplatin (12.5 µM), and an antagonism at high concentrations of cisplatin (25–50 µM). During pre-treatment of TOV-21G cells with morin for 24 h, followed by treatment with cisplatin alone for the next 24 h (APP:2; Fig. 2a), the most frequently observed interaction was antagonism. The exceptions were a synergism at the lowest dose of cisplatin (3.125 µM) and an additive effect at the cisplatin concentration of 12.5 µM. Pre-treatment of TOV-21G cells with morin for 24 h, followed by co-treatment with morin and cisplatin for another 24 h (APP:3; Fig. 2a) revealed strong antagonism (CI  >> 1.1) in each combination of morin–cisplatin concentrations.

Similarly to TOV-21G, when SK-OV-3 cells were treated with morin and cisplatin simultaneously for 24 h (APP:1; Fig. 2b), synergism was observed at low doses of cisplatin (3.125–6.25 µM) and low and medium doses of morin (100–200 µM). Also an additive effect occurred at the highest concentration of morin (250 µM) combined with low concentrations of cisplatin (3.125–6.25 µM). However, in contrast to TOV-21G, the additive effect was observed also in case of high doses of cisplatin (25–50 µM) and low doses of morin (100–150 µM). High concentrations of morin (200–250 µM) and cisplatin (25–50 µM) indicated antagonism, same as it was noticed in TOV-21G cells. Pre-treatment of SK-OV-3 cells with morin for 24 h, followed by treatment with cisplatin alone for the next 24 h (APP:2; Fig. 2b) revealed an antagonism in most morin–cisplatin ratios. On the contrary to TOV-21G, pre-treatment of SK-OV-3 cells with morin for 24 h, followed by co-treatment with morin and cisplatin for another 24 h (APP:3; Fig. 2b) seemed to be much more effective. Antagonism was observed only at high concentrations of cisplatin (25–50 µM). The rest combinations mostly revealed an additive effect.

### Morin boosts the anti-proliferative effect of cisplatin against ovarian cancer cells

Based on the results obtained from morin–cisplatin combination cytotoxicity study, in cell proliferation assay we decided to analyse only the selected concentrations of these drugs in optimal treatment approaches. Doses, which were chosen for the assessment, were generally those that exhibited the additive effect or synergism in morin–cisplatin combination cytotoxicity study.

**Fig. 3:**
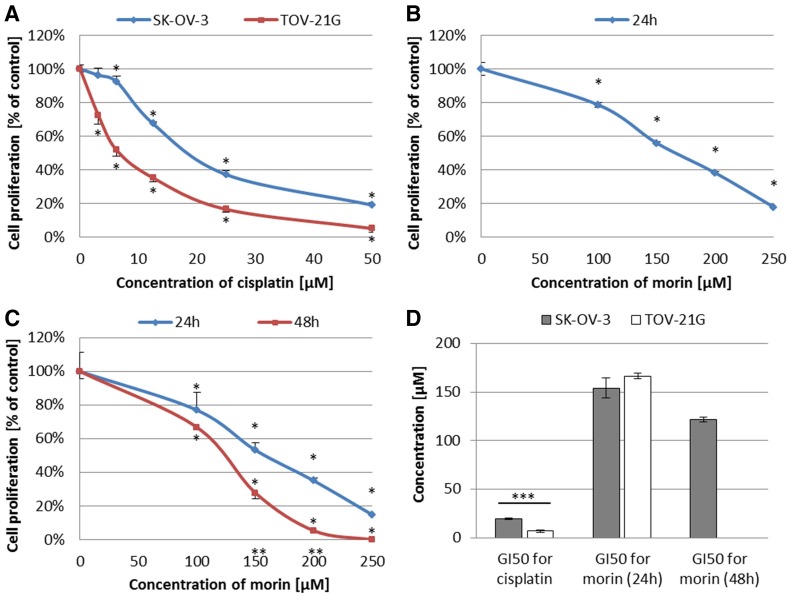
Effects of morin and cisplatin on proliferation of TOV-21G (cisplatin-sensitive) and SK-OV-3 (cisplatin-resistant) human ovarian cancer cells. The cells were treated with indicated concentrations of the drugs in indicated periods of time: **a** TOV-21G and SK-OV-3 cells treated with cisplatin (3.125–50 µM) for 24 h, **b** TOV-21G cells treated with morin (100–250 µM) for 24 h, **c** SK-OV-3 cells treated with morin (100–250 µM) for 24 h and 48 h. Cell proliferation was analysed by EdU incorporation assay. The data are shown as mean ± SD of triplicate experiments. Asterisk: ANOVA *p* < 0.05 between the cells treated with the different concentrations of drug and the untreated control group in the same period of time (dose-dependence). Double asterisk: ANOVA *p* < 0.05 between the cells treated with the same concentration of drug in the different period of time (time-dependence). The anti-proliferative effect of morin was dose- (in both cell lines) and time-dependent (in SK-OV-3 cells), and cisplatin was dose-dependent (in both cell lines). **d** Comparison of GI_50_ values obtained for TOV-21G and SK-OV-3 cells treated with morin for 24 h and/or 48 h or cisplatin for 24 h. Triple asterisk: *T* test *p* < 0.05 between GI_50_ values obtained for the same drug in the same period of time in different cell lines (TOV-21G or SK-OV-3)

First of all, we treated TOV-21G and SK-OV-3 cells with cisplatin (3.125–50 µM) for 24 h or morin (100–250 µM) for 24 h (TOV-21G and SK-OV-3) and 48 h (only SK-OV-3 cells, because in the case of TOV-21G cells, APP:3, which included 48 h treatment with morin, revealed strong antagonism between the both drugs). Cisplatin revealed an anti-proliferative effect against both cell lines in a dose-dependent manner (both *p* < 0.001; Fig. 3a). Similarly to IC_50_ values obtained from cell viability assay, the calculated GI_50_ values (GI_50_ = 19.73 ± 0.81 µM for SK-OV-3 and GI_50_ = 6.83 ± 1.35 µM for TOV-21G) were statistically different (*p* < 0.001; Fig. 3d). Moreover, morin exhibited an anti-proliferative activity against TOV-21G cells in a dose-dependent manner (*p* < 0.001; Fig. 3b) and against SK-OV-3 cells in a dose- and time-dependent manner (*p* < 0.001; Fig. 3c). As in the case of the cytotoxicity assay, also in this case there were no significant differences between GI_50_ values calculated for both cell lines during 24-h treatment (GI_50_ = 166.44 ± 3.13 µM and 154.21 ± 10.26 µM for TOV-21G and SK-OV-3, respectively). The GI_50_ value for SK-OV-3 cells treated with morin for 48 h was equalled 121.53 ± 2.37 µM.

**Fig. 4:**
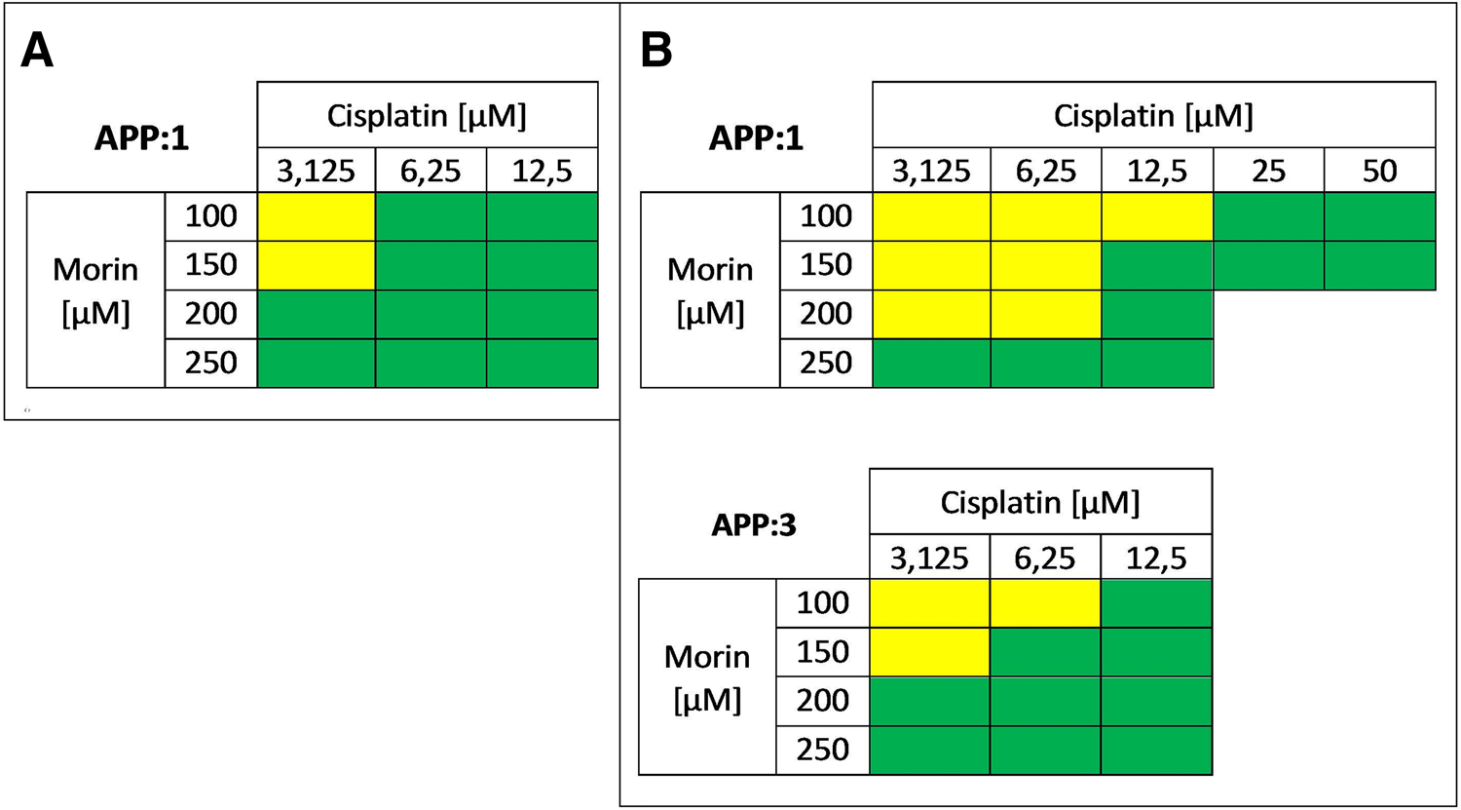
Anti-proliferative effect of morin–cisplatin combination on **a** TOV-21G (cisplatin-sensitive) and **b** SK-OV-3 (cisplatin-resistant) human ovarian cancer cells. The cells were treated with different combinations of morin–cisplatin according to selected approaches: APP:1—simultaneous treatment with both drugs for 24 h; APP:3—pre-treatment with morin for 24 h, followed by co-treatment with morin and cisplatin for another 24 h. After the treatments the cell proliferation was determined (EdU incorporation assay), followed by the calculation of combination index (CI). The CI values are shown as heat maps, where the effect of a drug combination is synergistic if CI < 0.9 (green colour); additive if 0.9 ≤ CI ≤ 1.1 (yellow colour); antagonistic if CI > 1.1 (red colour)

Second of all, we studied the effect of the morin–cisplatin combination on cell proliferation in order to select the most efficient concentrations of both drugs for further steps of this experiment. All *r* values calculated by CompuSyn software (ComboSyn, Inc.) were greater than 0.95, as it was described for the cell culture experiments [1, 24]. The results are shown as a heat map in Fig. 4. TOV-21G cells, treated with morin (3.125–12.5 µM) and cisplatin (100–250 µM) simultaneously for 24 h (APP:1; Fig. 4a), mostly revealed strong synergism (CI  << 0.9). In case of SK-OV-3 cells, treatment with morin (3.125–50 µM) and cisplatin (100–250 µM) simultaneously for 24 h (APP:1; Fig. 4b) generally revealed an additive effect at low cisplatin (3.125–6.25 µM) doses and a strong synergism (CI << 0.9) at medium and high cisplatin (12.5–50 µM) concentrations. During pre-treatment of SK-OV-3 cells with morin for 24 h, followed by co-treatment with cisplatin and morin for the next 24 h (APP:3; Fig. 4b), the most frequently noticed interaction was strong synergism (CI  << 0.9).

### Morin promotes cisplatin-induced apoptosis of ovarian cancer cells

In apoptosis assay we analysed the selected concentrations of morin and cisplatin in optimal treatment approaches, determined on the basis of the results obtained from morin–cisplatin combination cytotoxicity and proliferation study.

**Fig. 5:**
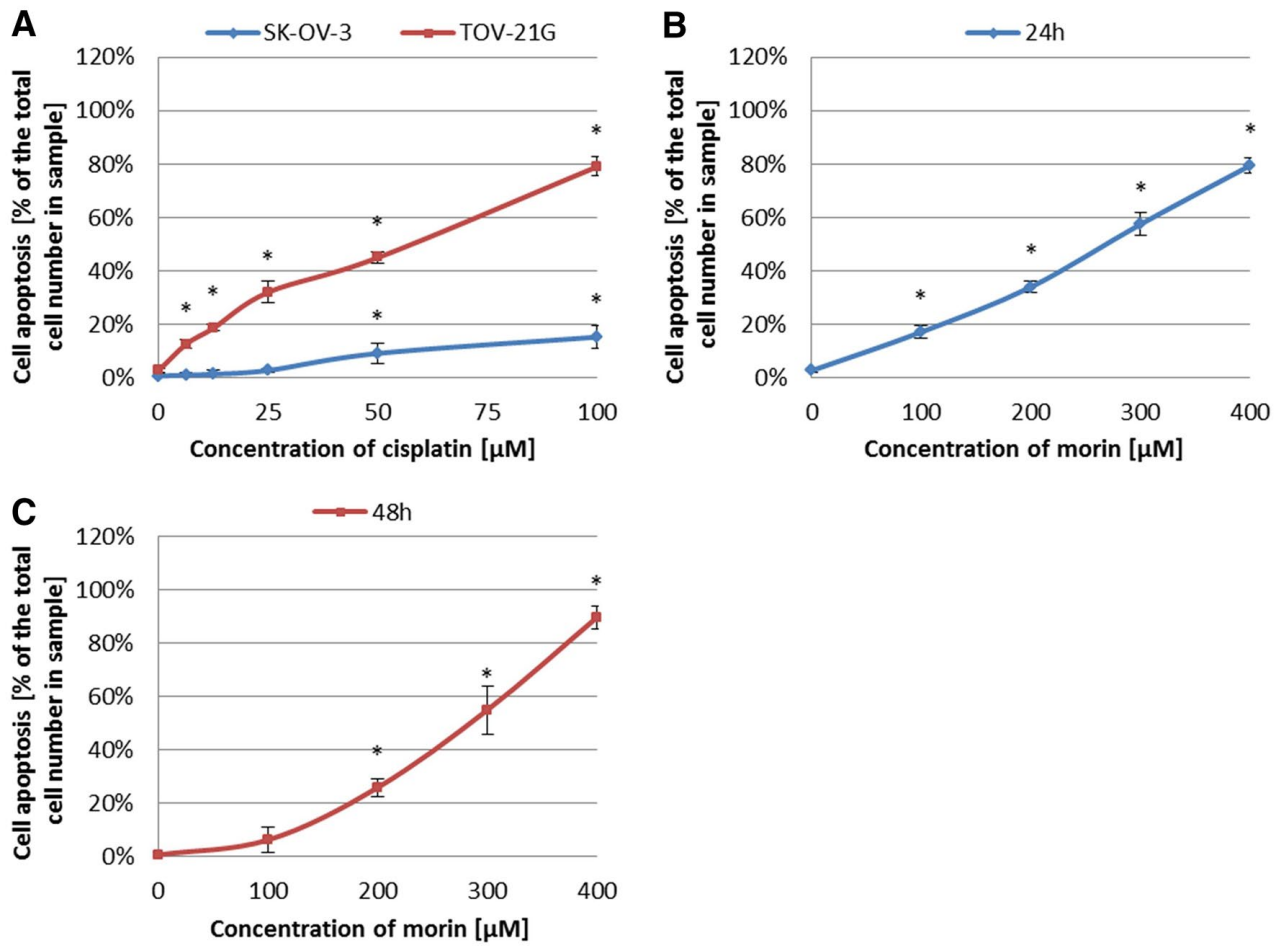
Effects of morin and cisplatin on apoptosis of TOV-21G (cisplatin-sensitive) and SK-OV-3 (cisplatin-resistant) human ovarian cancer cells. The cells were treated with indicated concentrations of the drugs in indicated periods of time: **a** TOV-21G and SK-OV-3 cells treated with cisplatin (6.25–100 µM) for 24 h, **b** TOV-21G cells treated with morin (100–400 µM) for 24 h, **c** SK-OV-3 cells treated with morin (100–400 µM) for 48 h. Cell apoptosis was analysed by FITC annexin V and PI staining. The data are shown as mean ± SD of triplicate experiments. Asterisk: ANOVA *p* < 0.05 between the cells treated with the different concentrations of drug and the untreated control group (dose-dependence). The pro-apoptotic effect of morin and cisplatin was dose-dependent in both cell lines

First, we treated TOV-21G and SK-OV-3 cells with cisplatin (6.25–100 µM) for 24 h or morin (100–400 µM) for 24 h (TOV-21G) or 48 h (SK-OV-3). Figure 5 shows that both morin and cisplatin significantly induced apoptosis of TOV-21G and SK-OV-3 cells in a dose-dependent manner (all *p* < 0.001). However, we found that the sensibility of TOV-21G to cisplatin was greater than that of SK-OV-3.

**Fig. 6:**
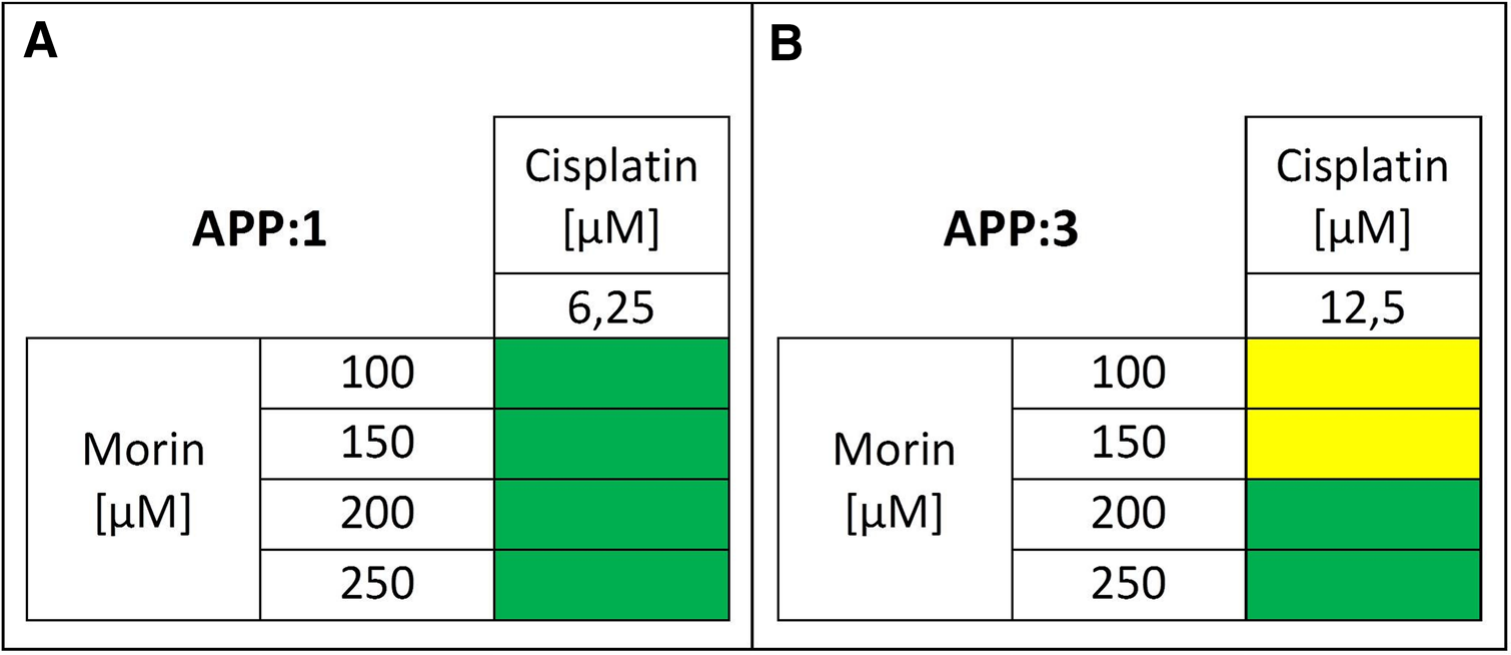
Pro-apoptotic effect of morin–cisplatin combination on **a** TOV-21G (cisplatin-sensitive) and **b** SK-OV-3 (cisplatin-resistant) human ovarian cancer cells. The cells were treated with different combinations of morin–cisplatin according to selected approaches: APP:1—simultaneous treatment with both drugs for 24 h; APP:3—pre-treatment with morin for 24 h, followed by co-treatment with morin and cisplatin for another 24 h. After the treatments the cell apoptosis was determined (FITC annexin V and PI staining), followed by the calculation of combination index (CI). The CI values are shown as heat maps, where the effect of a drug combination is synergistic if CI < 0.9 (green colour); additive if 0.9 ≤ CI ≤ 1.1 (yellow colour); antagonistic if CI > 1.1 (red colour)

Second, we evaluated the effect of the selected morin–cisplatin concentration combinations on cell apoptosis. All *r* values calculated by CompuSyn software (ComboSyn, Inc.) were greater than 0.95, as it was described for the cell culture experiments [1, 24]. The results are shown as a heat map in Fig. 6. The simultaneous treatment of TOV-21G with morin (100–250 µM) and cisplatin (6.25 µM) for 24 h (APP:1; Fig. 6a) revealed a synergism. In the case of SK-OV-3 cells, pre-treatment with morin for 24 h, followed by co-treatment with morin and cisplatin (12.5 µM) for another 24 h (APP:3; Fig. 6b), showed an additive effect at low morin doses (100–150 µM) and a synergistic effect at high morin (200–250 µM) concentrations.

### The sensitization of ovarian cancer cells on cisplatin is associated with decreased level of galectin-3 expression

To evaluate if the sensitization of TOV-21G and SK-OV-3 cells to cisplatin, caused by morin, is associated with changes in galectin-3 expression, we performed Real-Time™ RT-PCR analysis and ELISA assay. We tested selected and optimal treatment approaches, based on the previous steps of the experiment. TOV-21G cells were co-treated with morin (100–250 µM) and cisplatin (6.25 µM) for 24 h (APP:1). SK-OV-3 cells were pre-treated with morin (100–250 µM) for 24 h and then co-treated with morin (100–250 µM) and cisplatin (12.5 µM) for another 24 h (APP:3). In Real-Time™ RT-PCR analysis, the machine did not detect any amplification in no template controls (no *C*_q_ values were given by the software of the machine). The *r*^2^ values calculated for standard curves were greater than 0.995 in Bradford assay and 0.999 in ELISA assay.

**Fig. 7:**
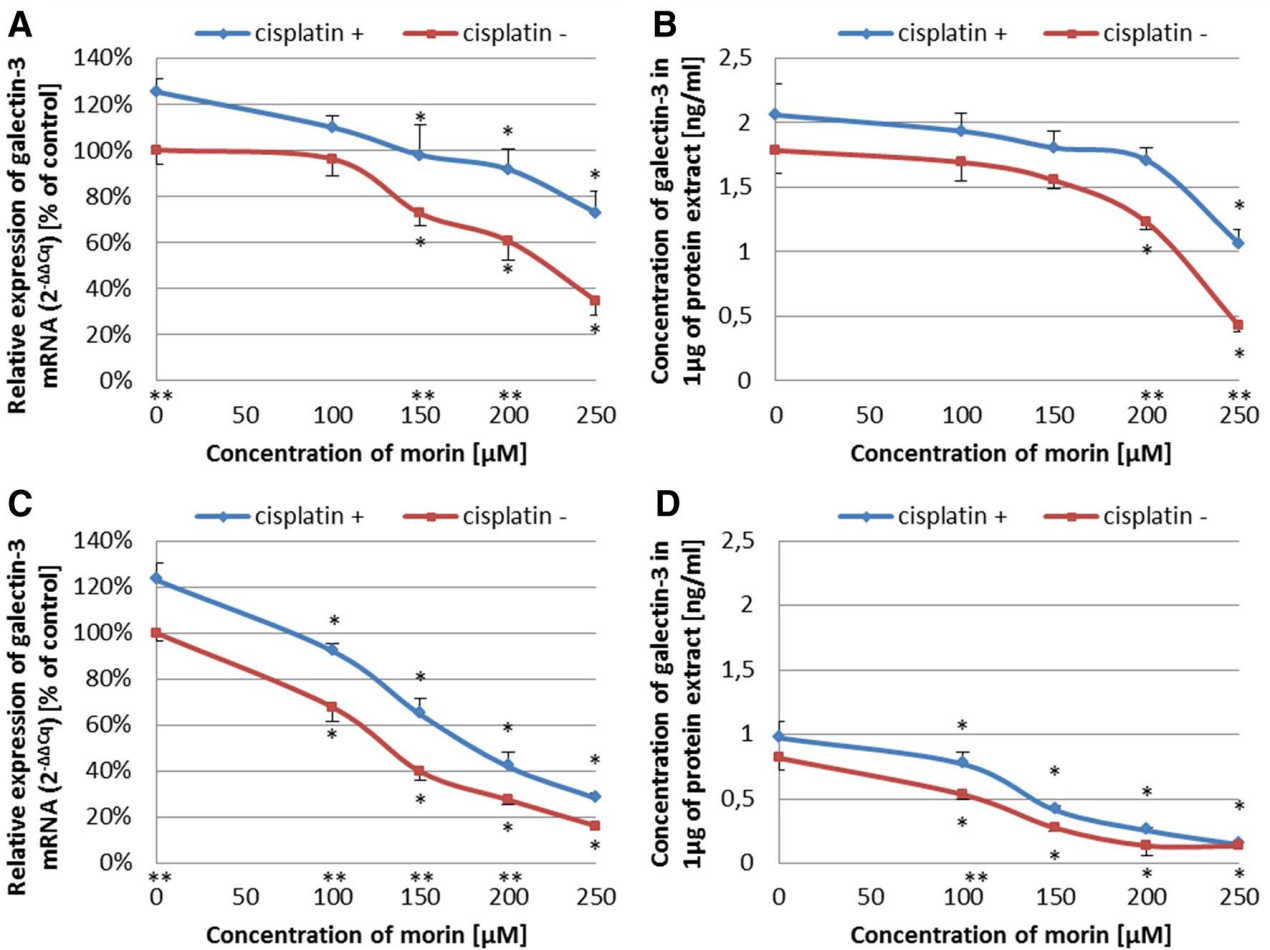
Effects of morin and cisplatin on the expression of galectin-3 at the mRNA and protein level in TOV-21G (cisplatin-sensitive) and SK-OV-3 (cisplatin-resistant) human ovarian cancer cells. The cells were treated with indicated concentrations of the drugs in indicated periods of time: **a**, **b** TOV-21G cells treated with morin (100–250 µM) and with or without cisplatin (6.25 µM) for 24 h (APP:1), **c**, **d** SK-OV-3 cells pre-treated with morin (100–250 µM) for 24 h and then treated with morin (100–250 µM) and with or without cisplatin (12.5 µM) for another 24 h (APP:3). At the mRNA level, the expression was analysed by Real Time™ RT-PCR technique. At the protein level, the expression was assessed by ELISA assay. The data are shown as mean ± SD of triplicate experiments. Asterisk: ANOVA *p* < 0.05 between the cells treated with the different concentrations of morin and the untreated control group (dose-dependence). Double asterisk: ANOVA *p* < 0.05 between the cells treated with the same concentration of morin in the presence or absence of cisplatin. Morin significantly reduced the expression of galectin-3 at mRNA and protein level in a dose-dependent manner in both cell lines. The presence of cisplatin significantly increased the level of galectin-3 at mRNA and protein level in both cell lines, without significantly affecting the ability of morin to reduce the expression of galectin-3

We found that morin at the concentration range of 100–250 µM significantly reduced the expression of galectin-3 at mRNA and protein level in both cell lines in a dose-dependent manner (all *p* < 0.001; Fig. 7). Interestingly, the effect of cisplatin was opposite, since it significantly increased the level of galectin-3 mRNA and protein in TOV-21G and SK-OV-3 cells (all *p* < 0.001; Fig. 7). However, the results also indicated that the presence of cisplatin did not significantly affect the ability of morin to reduce the expression of galectin-3 at both levels (all *p* > 0.05).

## Discussion

Ovarian cancer is one of the most lethal cancers of the female reproductive system worldwide. The early stages can be effectively treated with platinum-based chemotherapy (survival rates are more than 80%). However, most patients are diagnosed at late stage, what correlates with resistance to conventional platinum-based treatment and poor survival rates of approximately 20%. Therefore, there is an urgent need to discover novel therapeutic approaches to overcome drug resistance [25, 26]. Recently, there has been a growing interest in natural dietary phytochemicals, which could be co-administered with standard chemotherapeutic drugs in order to achieve better clinical response [26]. Increasing evidence suggests that many of these dietary compounds, such as capsaicin [1], curcumin [1, 27], thymoquinone [24], epigallocatechin gallate [26], methoxyphenyl chalcone [28], and delphinidin [29], sensitize ovarian cancer cells to cisplatin, when acting in combination. The last three of mentioned phytochemicals belong to flavonoids, which are secondary metabolites widely distributed among fruits and vegetables. Among flavonoids, there is also a subgroup of flavonols, which, according to published data, augment the activity of cisplatin against ovarian cancer cells. These flavonols are: hyperoside [4], kaempferol [30], and the best studied—quercetin [3, 24, 27, 31]. However, until now there has been no research on the use of morin, a structural isomer of quercetin, in ovarian cancer. In the present study, we for the first time demonstrate that morin possesses antitumor activity against ovarian cancer cells and that co-treatment of the cells with selected concentrations of morin and cisplatin reveals a synergism, which leads to sensitization of the cells to cisplatin by reducing cell viability and proliferation as well as increasing the induction of apoptosis. We also demonstrate that during this sensitization, morin significantly reduces the expression of galectin-3 at the mRNA and protein level regardless of the presence of cisplatin.

Morin (3,5,7,2′,4′-pentahydroxyflavone) is a flavonoid (flavonol), originally extracted from plants of Moraceae family [20, 21]. The anticancer effect of morin has been well known by inhibiting proliferation of cells of multiple tumours, such as leukaemia [20], squamous cell carcinoma [20], colorectal cancer [20, 32], and breast cancer [33]. Moreover, morin enhances apoptosis and chemo-sensitivity of prostate cancer cell lines to paclitaxel [21], and in combination with MST-312 sensitizes 5-fluorouracil (5-FU)-resistant human colorectal cancer cells for 5-FU [20]. Furthermore, morin derivatives potentiate the effects of vincristine in the treatment of adriamycin-resistant and -sensitive human myelogenous leukaemia [34]. These findings are consistent with our data that in TOV-21G (cisplatin-sensitive) and SK-OV-3 (cisplatin-resistant) ovarian cancer cell lines morin significantly supresses cell viability (TOV-21G *p* < 0.01; SK-OV-3 *p* < 0.001; Fig. 1b, c) and proliferation (both *p* < 0.001; Fig. 3b, c) in a dose- and time-dependent manner as well as promotes apoptosis (both *p* < 0.001; Fig. 5b, c) in a dose-dependent manner. We believe that our results also confirms the cisplatin-sensitivity of TOV-21G cells and the cisplatin-resistance of SK-OV-3 cells, based on the statistical difference between the IC_50_ and GI_50_ values calculated for cisplatin for both cell lines (both *p* < 0.001; Figs. 1d, 3d). Moreover, our data also show that despite the significantly different response of TOV-21G and SK-OV-3 cell lines to cisplatin, their sensibility to morin was comparable. The calculated IC_50_ and GI_50_ values for morin were not statistically different in both cell lines (both *p* > 0.05; Figs. 1d, 3d). These data are in agreement with those obtained for quercetin in pairs of cisplatin-sensitive and -resistant ovarian cancer cell lines: OV2008 (sensitive) and C13* (resistant) [3], as well as A2780 (sensitive) and A2780^cisR^ (resistant) [2, 24]. In contrast, another study showed that the IC_50_ value for quercetin in A2780^cisR^ is almost twofold greater than in A2780 [1].

We also performed extensive drug combination studies based on cell viability, proliferation, and apoptosis assays, to select optimal treatment approaches. We found that in TOV-21G (cisplatin-sensitive) cells the additive or synergistic effects between morin and cisplatin occurred, when cells were treated with both drugs simultaneously for 24 h (APP:1; Figs. 2a, 4a, 6a), especially at low cisplatin concentrations (3.125–12.5 µM). On the contrary, the pre-treatment with morin for 24 h, followed by treatment with cisplatin alone for the next 24 h (APP:2; Fig. 2a), or the pre-treatment with morin for 24 h, followed by co-treatment with morin and cisplatin for another 24 h (APP:3; Fig. 2a) mostly revealed the antagonism. In SK-OV-3 (cisplatin-resistant) cells, we also observed the additive effect and the synergism in APP:1 (Figs. 2b, 4b), but the effects occurred not only at low concentrations of cisplatin, but in case of all tested doses (3.125–50 µM). However, at high cisplatin concentrations, the effects were observed only in combination with low doses of morin (100–150 µM). Furthermore, we also noticed these effects in APP:3 (Figs. 2b, 4b, 6b), especially at low cisplatin concentrations (3.125–12.5 µM). On the other hand, similarly to TOV-21G, APP:2 (Fig. 2b) also revealed mostly antagonism. Briefly, we believe that in the SK-OV-3 (cisplatin-resistant) cell line the additive and synergistic effects are noticeable in a broader range of doses of cisplatin, than in the TOV-21G (cisplatin-sensitive) cells. Moreover, in SK-OV-3 these effects occur not only in APP:1 (as it is in case of TOV-21G), but also in APP:3.

Analogous results, obtained for the combination of cisplatin and quercetin in SK-OV-3 ovarian cancer cells by other researchers, are partially consistent with ours. According to the published data, quercetin increased sensitivity of SK-OV-3 ovarian cancer cells to cisplatin, when it was added simultaneously with cisplatin (APP:1), as well as when it was pre-administrated for 24 h (APP:2) [27]. This superficial incompatibility in APP:2 can be simply explained. The authors treated cells with only one dose of cisplatin that equalled 2 µg/ml (approximately 6.67 µM). Although generally we observed the antagonism in APP:2 (Fig. 2b), we also noticed the synergism at the lowest concentration of morin (100 µM) and cisplatin (< 6.25 µM). In contrast, another study revealed that simultaneous treatment with quercetin and cisplatin for 4 days did not significantly increase the sensitivity of SK-OV-3 cells to the second drug. However, it could be explained by a very low concentration of cisplatin administrated to the cells by the authors (16.66 × 10^−5^ µM), even though the experiment was performed for 4 days [31].

Our results may raise the question about the potential cause of the differences in the cellular response between TOV-21G and SK-OV-3 to the treatment. This might be partially explained by the mechanism of action of morin and a various nature of both cell lines. First of all, the antitumor effect of morin lies in the repression of NF-κB activation, by inhibition of IKK (IκBα kinase) [19]. NF-κB is a heterodimer (p50/p65), which is sequestered in the cytoplasm in an inactive form by IκBα inhibitory subunit. After stimulation, e.g. by cisplatin, IKK complex (comprising of IKKα, IKKβ and IKKγ isoforms) is activated, what leads to IκBα phosphorylation by IKKβ. Finally, this results in ubiquitination and proteasomal degradation of IκBα and subsequent translocation of p50/p65 heterodimer to nucleus, where it regulates the expression of downstream genes [35]. It is well known that in SK-OV-3 ovarian cancer cell line, NF-κB is constitutively active [11]. On the other hand, it was found that SK-OV-3 has high baseline level of IKKβ, while TOV-21G has low baseline level of IKKβ [36]. Moreover, in cisplatin-resistant cells of head and neck squamous cell carcinoma, NF-κB/IKKβ signalling is up-regulated [35]. Maybe, the constitutive activation of NF-κB is caused by the high level of IKKβ, which is also a mediator of cellular response to cisplatin. Second , this is not the only possible action of morin inside cells. This flavonol also acts as an antioxidant, which reduces oxidative stress [7, 37]. The activity may be achieved due to the presence of the hydroxyl groups attached at various positions in its characteristic flavonoid structure, which can directly scavenge the reactive species [38]. Nonetheless, the anti-oxidative nature of morin results in sparing of cellular antioxidants, such as glutathione and in vivo study showed that pre-treatment with morin increased tissue GSH level [37, 38]. Moreover, an increased glutathione (a nucleophilic molecule) concentration neutralizes cisplatin before its binding with the DNA, and this leads to cisplatin-resistance [39]. As to the question about the cause of different outcomes in our drug combination studies, it appears that the low baseline level of IKKβ in TOV-21G compared to SK-OV-3 (according to [36]), may increase the anti-oxidative activity of morin, due to reduced amount of its molecular target (IKKβ). Perhaps in TOV-21G cells, simultaneous treatment with cisplatin and morin (APP:1) moved the balance of morin activity from anti-oxidative to IKK-inhibitive. However, after 24 h pre-treatment (APP:2 and APP:3), the cells were less vulnerable to cisplatin, what resulted in worse cellular response in APP:2 compared to APP:1. Moreover, co-administration of morin and cisplatin for another 24 h (APP:3), caused even further insensitivity to cisplatin. This idea could also clarify the outcomes obtained in SK-OV-3 cells. Maybe, APP:2 gave the worse results, because overcoming drug resistance, caused by high baseline level of IKKβ, can be achieved only during co-treatment with morin and cisplatin (APP:1 and APP:3). Furthermore, since the baseline level of IKKβ is high, in APP:3 morin did not have an opportunity to act like the antioxidant. Nonetheless, it should be noticed that the above explanation might be too simplistic, due to the complexity of the situation, caused by the involvement of multiple pathways associated with drug resistance.

Since we demonstrated that the combination of selected concentrations of morin and cisplatin sensitizes ovarian cancer cells to cisplatin and we also mentioned the importance of NF-κB pathway in cellular response for morin, it should not be a surprise that we decided to evaluate whether the sensitization of the cells is associated with the changes in the expression of an anti-apoptotic protein, regulated by NF-κB transcription factor [13]. This protein was galectin-3, which exhibits similarities to BCL-2 family through sharing the NWGR (N, asparagine; W, tryptophan; G, glycine; R, arginine) anti-death motif [16–18]. Up-regulation of galectin-3 has been noticed in many cancers (however, in some occurs down-regulation), including ovarian cancer [5, 15–18]. It is also well known that overexpression of galectin-3 in ovarian cancer is involved in drug resistance to cisplatin and paclitaxel [5, 15, 25]. Moreover, depletion of galectin-3 expression by siRNA enhances sensitivity of ovarian cancer cells to paclitaxel (in SK-OV-3 cells) [5, 25] and cisplatin [15], as well osteosarcoma cells to cisplatin [14], pancreatic cancer cells to cisplatin and gemcitabine [16], cholangiocarcinoma cells to cisplatin and 5-FU [18], anaplastic thyroid carcinoma cells to cisplatin [40], and prostate cancer to cisplatin [41]. However, there were also some attempts to decrease galectin-3 expression and sensitize cancer cells to chemotherapeutic agents by natural dietary phytochemicals. For example, modified citrus pectin (MCP), which is a non-toxic polysaccharide galectin-3 antagonist, augments prostate cancer cells to cisplatin [41]. Moreover, inhibition of galectin-3 by MCP sensitizes SK-OV-3 ovarian cancer cells to paclitaxel [25]. These data are consistent with our findings that during sensitization of TOV-21G and SK-OV-3 cells to cisplatin by morin, there is a significant reduction in galectin-3 expression at the mRNA and protein levels in both cell lines in a dose-dependent manner (all *p* < 0.001; Fig. 7). Surprisingly, our results also suggest that cisplatin significantly increases the level of galectin-3 mRNA and protein in both cell lines (all *p* < 0.001; Fig. 7); nonetheless, it does not significantly affect the ability of morin to reduce the expression of galectin-3 at both levels (all *p* > 0.05; Fig. 7). These data are similar to that of others, which shows that cisplatin effectively increases galectin-3 expression, what protects K562 human leukaemia cells from apoptosis [42, 43].

To summarize, we believe that we demonstrated the potential of morin as candidate for combination treatment of ovarian cancer. It should also be noticed that morin has certain advantages, which makes it an even more attractive candidate for use in therapies. Morin has found to be more active in blocking NF-κB activation than other flavonols such as quercetin and kaempferol [44]. Moreover, compared to morin, curcumin (another well-known inhibitor of NF-κB) has poor bioavailability and solubility in water, and so, its distribution in the body is limited [10]. In addition, morin appears to have no cytotoxic effect on normal cells, even at higher concentrations. For example, the drug did not affect the viability of MDA-MB-231 breast cancer cells and EA.hy 926 human umbilical endothelial cells at doses lower than 100 µM. However, at higher concentrations (100–200 µM), it reduced the viability of MDA-MB-231, and surprisingly also increased the viability of EA.hy 926 [33]. One more example is another experiment, in which the viability of RAW264.7 macrophage cells was not significantly altered during 24 h treatment with morin at up to the concentration as high as 500 μM [45].

## Conclusion

In this paper, we demonstrated that morin sensitizes TOV-21G (cisplatin-sensitive) and SK-OV-3 (cisplatin-resistant) ovarian cancer cells to cisplatin, what is associated with a decrease of the expression of galectin-3 (an anti-apoptotic protein). Our findings could make morin a useful candidate for oncological combination treatment with cisplatin. Moreover, the clinical application of cisplatin is limited by its dose-dependent severe side effects in normal tissues, such as nephrotoxicity, hepatotoxicity, neurotoxicity, ototoxicity and allergic reactions [3, 7, 24]. Compensation of these adverse effects can be achieved by combination treatment with a drug, which would reduce the systemic toxicity by acting as an antioxidant or by allowing to decrease the required dose of chemotherapeutic agent [24]. In fact, it has been proven that morin treatment along with cisplatin significantly mitigated the negative influence of cisplatin on liver [37] and kidney function [7, 37, 38], through the reduction of oxidative stress, inflammation and apoptosis [7]. On the other hand, we (along with many others) have demonstrated that morin sensitizes various cancer cells to chemotherapeutic agents, what may allow to decrease the required doses in clinical treatment in the future.

## References

[1] Arzuman L, Beale P, Chan C (2014). Synergism from combinations of tris (benzimidazole) monochloroplatinum(II) chloride with capsaicin, quercetin, curcumin and cisplatin in human ovarian cancer cell lines. Anticancer Res.

[2] Long Q, Xie Y, Huang Y (2013). Induction of apoptosis and inhibition of angiogenesis by PEGylated liposomal quercetin in both cisplatin-sensitive and cisplatin-resistant ovarian cancers. J Biomed Nanotechnol.

[3] Yang Z, Liu Y, Liao J (2015). Quercetin induces endoplasmic reticulum stress to enhance cDDP cytotoxicity in ovarian cancer: involvement of STAT3 signaling. FEBS J.

[4] Zhu X, Ji M, Han Y (2017). PGRMC1-dependent autophagy by hyperoside induces apoptosis and sensitizes ovarian cancer cells to cisplatin treatment. Int J Oncol.

[5] Kang HG, Kim S, Cho Y (2016). Galectin-3 supports stemness in ovarian cancer stem cells by activation of the Notch1 intracellular domain. Oncotarget.

[6] Koukoura O, Spandidos DA, Daponte A, Sifakis S (2014). DNA methylation profiles in ovarian cancer: implication in diagnosis and therapy (review). Mol Med Rep.

[7] Wei Z, He X, Kou J (2015). Renoprotective mechanisms of morin in cisplatin-induced kidney injury. Int Immunopharmacol.

[8] Davis A, Tinker A, Friedlander M (2014). “Platinum resistant” ovarian cancer: what is it, who to treat and how to measure benefit?. Gynecol Oncol.

[9] Galluzzi L, Vitale I, Michels J (2014). Systems biology of cisplatin resistance: past, present and future. Cell Death Dis.

[10] Oiso S, Ikeda R, Nakamura K (2012). Involvement of NF-κB activation in the cisplatin resistance of human epidermoid carcinoma KCP-4 cells. Oncol Rep.

[11] Ataie-Kachoie P, Badar S, Morris DL, Pourgholami MH (2013). Minocycline targets the NF-κB nexus through suppression of TGF-β1-TAK1-IκB signaling in ovarian cancer. Mol Cancer Res.

[12] Mabuchi S, Ohmichi M, Nishio Y (2004). Inhibition of NFκB increases the efficacy of cisplatin in in vitro and in vivo ovarian cancer models. J Biol Chem.

[13] Wang L, Guo X-L (2016). Molecular regulation of galectin-3 expression and therapeutic implication in cancer progression. Biomed Pharmacother.

[14] Bin Park G, Kim DJ, Kim YS (2015). Silencing of galectin-3 represses osteosarcoma cell migration and invasion through inhibition of FAK/Src/Lyn activation and β-catenin expression and increases susceptibility to chemotherapeutic agents. Int J Oncol.

[15] Oishi T, Itamochi H, Kigawa J (2007). Galectin-3 may contribute to cisplatin resistance in clear cell carcinoma of the ovary. Int J Gynecol Cancer.

[16] Kobayashi T, Shimura T, Yajima T (2011). Transient silencing of galectin-3 expression promotes both in vitro and in vivo drug-induced apoptosis of human pancreatic carcinoma cells. Clin Exp Metastasis.

[17] Pokrywka M, Bubka M, Janik M (2016). Gal-3 does not suppress cisplatin-induced apoptosis in A-375 melanoma cells. Cell Biol Int.

[18] Wongkham S, Junking M, Wongkham C (2009). Suppression of galectin-3 expression enhances apoptosis and chemosensitivity in liver fluke-associated cholangiocarcinoma. Cancer Sci.

[19] Venu Gopal J (2013). Morin hydrate: botanical origin, pharmacological activity and its applications: a mini-review. Pharmacogn J.

[20] Chung SS, Oliva B, Dwabe S, Vadgama JV (2016). Combination treatment with flavonoid morin and telomerase inhibitor MST-312 reduces cancer stem cell traits by targeting STAT3 and telomerase. Int J Oncol.

[21] Li B, Jin X, Meng H (2017). Morin promotes prostate cancer cells chemosensitivity to paclitaxel through miR-155/GATA3 axis. Oncotarget.

[22] Chou TC (2010). Drug combination studies and their synergy quantification using the Chou–Talalay method. Cancer Res.

[23] Bijnsdorp IV, Giovannetti E, Peters GJ (2011). Analysis of drug interactions. Methods Mol Biol.

[24] Nessa MU, Beale P, Chan C (2011). Synergism from combinations of cisplatin and oxaliplatin with quercetin and thymoquinone in human ovarian tumour models. Anticancer Res.

[25] Hossein G, Keshavarz M, Ahmadi S, Naderi N (2013). Synergistic effects of PectaSol-C modified citrus pectin an inhibitor of galectin-3 and paclitaxel on apoptosis of human SKOV-3 ovarian cancer cells. Asian Pac J Cancer Prev.

[26] Mazumder MEH, Beale P, Chan C (2012). Epigallocatechin gallate acts synergistically in combination with cisplatin and designed trans-palladiums in ovarian cancer cells. Anticancer Res.

[27] Chan MM, Fong D, Soprano KJ, Holmes WF (2002). Inhibition of growth and sensitization to cisplatin-mediated killing of ovarian cancer cells by polyphenolic chemopreventive agents. J Cell Physiol.

[28] Su Y-K, Huang W-C, Lee W-H (2017). Methoxyphenyl chalcone sensitizes aggressive epithelial cancer to cisplatin through apoptosis induction and cancer stem cell eradication. Tumour Biol.

[29] Lim W, Jeong W, Song G (2016). Delphinidin suppresses proliferation and migration of human ovarian clear cell carcinoma cells through blocking AKT and ERK1/2 MAPK signaling pathways. Mol Cell Endocrinol.

[30] Luo H, Daddysman MK, Rankin GO (2010). Kaempferol enhances cisplatin’s effect on ovarian cancer cells through promoting apoptosis caused by down regulation of cMyc. Cancer Cell Int.

[31] Maciejczyk A, Surowiak P (2013). Quercetin inhibits proliferation and increases sensitivity of ovarian cancer cells to cisplatin and paclitaxel. Ginekol Pol.

[32] Hyun HB, Lee WS, Go SI (2015). The flavonoid morin from Moraceae induces apoptosis by modulation of Bcl-2 family members and Fas receptor in HCT 116 cells. Int J Oncol.

[33] Jin H, Lee WS, Eun SY (2014). Morin, a flavonoid from Moraceae, suppresses growth and invasion of the highly metastatic breast cancer cell line MDA-MB-231 partly through suppression of the Akt pathway. Int J Oncol.

[34] Ikegawa T, Ohtani H, Koyabu N (2002). Inhibition of P-glycoprotein by flavonoid derivatives in adriamycin-resistant human myelogenous leukemia (K562/ADM) cells. Cancer Lett.

[35] Li Z, Yang Z, Lapidus RG (2015). IKK phosphorylation of NF-κB at serine 536 contributes to acquired cisplatin resistance in head and neck squamous cell cancer. Am J Cancer Res.

[36] Son D-S, Kabir SM, Dong Y (2013). Characteristics of chemokine signatures elicited by EGF and TNF in ovarian cancer cells. J Inflamm.

[37] Kv A, Madhana RM, Kasala ER (2016). Morin hydrate mitigates cisplatin-induced renal and hepatic injury by impeding oxidative/nitrosative stress and inflammation in mice. J Biochem Mol Toxicol.

[38] Kaltalioglu K, Coskun-Cevher S (2016). Potential of morin and hesperidin in the prevention of cisplatin-induced nephrotoxicity. Ren Fail.

[39] Samuel P, Pink RC, Brooks SA, Carter DR (2016). MiRNAs and ovarian cancer: a miRiad of mechanisms to induce cisplatin drug resistance. Expert Rev Anticancer Ther.

[40] Lavra L, Ulivieri A, Rinaldo C (2009). Gal-3 is stimulated by gain-of-function p53 mutations and modulates chemoresistance in anaplastic thyroid carcinomas. J Pathol.

[41] Wang Y, Nangia-Makker P, Balan V (2010). Calpain activation through galectin-3 inhibition sensitizes prostate cancer cells to cisplatin treatment. Cell Death Dis.

[42] Tseng PC, Chen CL, Shan YS, Lin CF (2016). An increase in galectin-3 causes cellular unresponsiveness to IFN-γ-induced signal transduction and growth inhibition in gastric cancer cells. Oncotarget.

[43] Cheng YL, Huang WC, Chen CL (2011). Increased galectin-3 facilitates leukemia cell survival from apoptotic stimuli. Biochem Biophys Res Commun.

[44] Manna SK, Aggarwal RS, Sethi G (2007). Morin (3,5,7,2′,4′ pentahydroxyflavone) abolishes nuclear factor-κB activation induced by various carcinogens and inflammatory stimuli, leading to suppression of nuclear factor-κB regulated gene expression and up regulation of apoptosis. Clin Cancer Res.

[45] Dhanasekar C, Kalaiselvan S, Rasool M (2015). Morin, a bioflavonoid suppresses monosodium urate crystal-induced inflammatory immune response in RAW 264.7 macrophages through the inhibition of inflammatory mediators, intracellular ROS levels and NF-κB activation. PLoS ONE.

